# Supplementary material for: Extension of Lung Damage at Chest Computed Tomography in Severely Ill COVID-19 Patients Treated with Interleukin-6 Receptor Blockers Correlates with Inflammatory Cytokines Production and Prognosis
Source: Tomography. 2023 May 11;9(3):981–94. doi: 10.3390/tomography9030080 (PMC10204376; doi:10.3390/tomography9030080)
Supplement: Supplementary file 1 [file tomography-09-00080-s001.zip › tomography-2360685-supplementary.pdf]

Criteria for definition of Cytokine storm

Laboratory results at baseline have been used to define the presence of CS as defined by published criteria in COVID-19 pneumonia: ferritin >250 ng/mL, CRP >4.6mg/dL, and at least one abnormality in three clusters of laboratory variables. The first cluster included albumin <2.8 g/dL, lymphocytes <10.2%, or neutrophiles >11400/mm^3, the second cluster included alanine aminotransferase >60U/l, aspartate aminotransferase >87 U/l, D-dimers >4930 ng/mL, LDH >416 U/l or Troponin I >1.09 ng/ml, and the third cluster included anion gap<6.8 mmol/l, chloride >106 mmol/l, potassium >4.9 mmol/l, or blood urea nitrogen/creatinine ratio >29.
